# Cotton-Net: efficient and accurate rapid detection of impurity content in machine-picked seed cotton using near-infrared spectroscopy

**DOI:** 10.3389/fpls.2024.1334961

**Published:** 2024-01-25

**Authors:** Qingxu Li, Wanhuai Zhou, Xuedong Zhang, Hao Li, Mingjie Li, Houjun Liang

**Affiliations:** ^1^ College of Computer Science, Anhui University of Finance & Economics, Bengbu, China; ^2^ Institute of Cotton Engineering, Anhui University of Finance & Economics, Bengbu, China

**Keywords:** seed cotton, impurity, cotton-net, near-infrared spectroscopy, rapid detection

## Abstract

Widespread adoption of machine-picked cotton in China, the impurity content of seed cotton has increased significantly. This impurity content holds direct implications for the valuation of seed cotton and exerts a consequential influence on the ensuing quality of processed lint and textiles. Presently, the primary approach for assessing impurity content in seed cotton primarily depends on semi-automated testing instruments, exhibiting suboptimal detection efficiency and not well-suited for the impurity detection requirements during the purchase of seed cotton. To address this challenge, this study introduces a seed cotton near-infrared spectral (NIRS) data acquisition system, facilitating the rapid collection of seed cotton spectral data. Three pretreatment algorithms, namely SG (Savitzky-Golay convolutional smoothing), SNV (Standard Normal Variate Transformation), and Normalization, were applied to preprocess the seed cotton spectral data. Cotton-Net, a one-dimensional convolutional neural network aligned with the distinctive characteristics of the seed cotton spectral data, was developed in order to improve the prediction accuracy of seed cotton impurity content. Ablation experiments were performed, utilizing SELU, ReLU, and Sigmoid functions as activation functions. The experimental outcomes revealed that after normalization, employing SELU as the activation function led to the optimal performance of Cotton-Net, displaying a correlation coefficient of 0.9063 and an RMSE (Root Mean Square Error) of 0.0546. In the context of machine learning modeling, the LSSVM model, developed after Normalization and Random Frog algorithm processing, demonstrated superior performance, achieving a correlation coefficient of 0.8662 and an RMSE of 0.0622. In comparison, the correlation coefficient of Cotton-Net increased by 4.01%. This approach holds significant potential to underpin the subsequent development of rapid detection instruments targeting seed cotton impurities.

## 1 Introduction

China is a major cotton-producing nation, with seed cotton production exceeding 16 million tons over the past five years (Lu et al., 2022). Notably, the Xinjiang region commands approximately 85% of the nation’s cotton production, and within this landscape, the prevalence of mechanically harvested cotton has surged to over 80% (Zhang et al., 2022). The level of impurity in machine-picked seed cotton significantly exceeds that in hand-picked seed cotton (Singh et al., 2022). Generally, the impurity content in machine-picked seed cotton ranges from 8 to 16%, in contrast to the approximate 3% observed in hand-picked seed cotton. The presence of impurities in seed cotton has far-reaching consequences, affecting not only the processing of lint cotton but also the final quality of downstream textile products (Zhou et al., 2016). Given the widespread cultivation of machine-picked cotton, identifying and mitigating seed cotton impurities has become increasingly important. Furthermore, the impurity content present in seed cotton bears a direct correlation with the market value of the cotton. Generally, a lower impurity content corresponds to a higher trading price. At present, the prevailing method for assessing seed cotton impurity content involves the utilization of semi-automated testing instruments. Nonetheless, these instruments exhibit unwieldiness, sluggishness, and consume considerable time, with a single cotton sample necessitating 20 to 30 minutes for assessment. Evidently, these instruments are ill-suited for on-site impurity content detection at seed cotton purchase locations. Consequently, a pressing need emerges for swift seed cotton impurity content detection technologies within the contemporary cotton production and processing paradigm. Such advancements hold profound significance in propelling China’s cotton industry toward intelligent and automated growth, while concurrently safeguarding the interests of cotton farmers.

Seed cotton impurities are mostly composed of plant-based impurities and foreign fibers. Notably, the proportion of foreign fibers within these impurities is significantly lower than that of plant-based impurities, thus exerting a comparatively lesser influence on the overall impurity content of seed cotton. In the current landscape, a greater concentration of research endeavors are directed towards the detection of foreign fibers within cotton. Various technical methods, including machine vision (Zhang et al., 2011; Zhang and Li, 2014), hyperspectral imaging (Liu et al., 2022), and near-infrared spectroscopy (Du et al., 2023), have been harnessed to detect foreign fibers present in both lint and seed cotton samples. Researchers have also delved into studies focusing on the identification of non-foreign fiber impurities, specifically plant-based impurities, within both seed and lint cotton. These investigations have predominantly harnessed technical tools such as machine vision and spectroscopy techniques. Xu et al. (2023) employed machine vision in conjunction with the lightweight YOLOV4 algorithm to achieve impurity detection in lint cotton, yielding an impressive detection accuracy of 98.00%. Zhang et al. (2021; Zhang et al., 2022) harnessed image processing and the YOLOV4 algorithm to discern impurities within seed cotton, subsequently estimating impurity content through pixel area calculations. This approach yielded a noteworthy prediction accuracy of approximately 0.8 for impurity content. Chang et al. (2021) leveraged spectral data alongside machine learning algorithms to classify impurities in seed cotton, culminating in the collection of hyperspectral information from the seed cotton samples. This concerted effort yielded an impressive identification accuracy of 83.40%. Fortier et al. (2011) employed Fourier transform near-infrared spectroscopy to proficiently classify and identify impurities within lint cotton, achieving a remarkable identification accuracy of 97%. Liu and Foulk (2013) applied visible near-infrared spectroscopy to effectively classify leaf content in lint cotton, demonstrating a high classification accuracy of 95% within the spectral band spanning 1105 ~ 1700 nm. Gaitán-Jurado et al. (2008) employed near-infrared spectroscopy to ascertain both the moisture and impurity content of seed cotton. Notably, the moisture content displayed a standard deviation of 0.44%, encompassing a range of 5.38% to 14.96%. Furthermore, the classification of seed cotton samples based on impurity content achieved an accuracy rate of 80%.

NIRS is a technique that achieves precise and rapid determination of the content of one or more constituents in a target substance. This is accomplished by illuminating a sample under study with near-infrared light and subsequently analyzing the pertinent information inherent in the substance’s transmission or reflection of the light (Prananto et al., 2021). Zumba and Rogers (2016) utilized near-infrared spectroscopy to quantify the macronutrient value in cotton fibers. Rodgers and Beck (2009) and Yang et al. (2022) conducted research that focused on the utilization of NIRS for detecting cotton content within fabrics. Li et al. (2020) employed NIRS for the discrimination between plant-dyed and chemically dyed cotton. Suarez et al. (2017) conducted research focused on the detection of herbicide levels in cotton utilizing visible-near infrared spectral data. Du et al. (2022) employed NIRS in conjunction with convolutional neural networks to successfully discern used textiles.

Evidently, a considerable portion of the preceding investigations pertaining to impurity detection in both seed and lint cotton predominantly adopt a qualitative approach. Specifically, these studies primarily revolve around the identification of impurities, neglecting the quantification of impurity content within the seed cotton samples. Notably, Zhang et al. (2021, Zhang et al., 2022) stand out as the sole contributors who employed a visual method for determining the impurity content of seed cotton. Nevertheless, it remains apparent that there exists potential for enhancing the precision of the detection outcomes. It is feasible to use NIRS for the evaluation of cotton quality and the detection of impurities. In this study, the quantification of impurity content in seed cotton is accomplished through the establishment of a spectral data acquisition system tailored for seed cotton impurities. The employed techniques encompass Cotton-Net and machine learning.

## 2 Methods and materials

### 2.1 Sample preparation

Forty-six kilogram of machine-picked seed cotton from Kuitun, Xinjiang, was prepared for experimentation. A total of 230 test samples were crafted, each comprising 200 g of machine-picked seed cotton. The samples were numbered for accurate identification. The precision and consistency of the weighing process were ensured by employing an electronic balance manufactured by HZ Electronic Technology Company. The electronic balance model used is HZY-B, with a minimum testing accuracy of 0.01g. Subsequent to the sample preparation, the seed cotton specimens were subjected to a controlled environment within a constant temperature and humidity chamber. The specimens were exposed to a stable temperature of 20 ± 1° and relative humidity of 60 ± 5%RH for a duration of 24 hours.

Seed cotton impurity quantification is conducted through the subsequent methodology: Initial separation involves segregating larger impurities from the cotton fibers via a sawtooth cotton gin. The resulting larger impurities are precisely weighed using an electronic balance. Furthermore, the Y101 impurity analyzer (manufactured by Shanghai Yuanqi Inspection Instrument Company, the roller has a diameter of 57.15mm.) is deployed to address the evaluation of smaller impurities during the ginning process of seed cotton. This specialized equipment facilitates the separation and subsequent weighing of minute impurities. By collectively considering the mass data garnered from both the larger and smaller impurities, a comprehensive assessment of the seed cotton’s impurity content is ascertained.

### 2.2 Spectral data acquisition system

**Figure 1:**
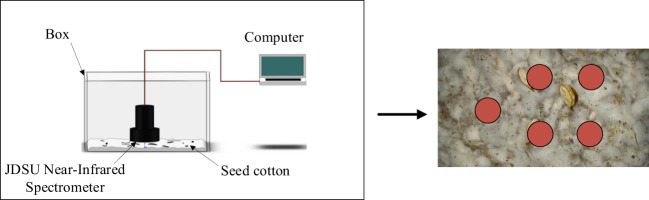
Spectral data acquisition system.

The seed cotton spectral data acquisition system (**Figure 1**) comprises a box, a computer, and a JDSU near-infrared spectrometer (model: JDSU MicroNIR) with a wavelength range spanning 950~1650 nm. This spectrometer features a built-in double-integrated vacuum tungsten lamp, offering a wavelength range for vacuum tungsten lamps from 200 nm to 2500 nm. The box is constructed from stainless steel metal material. The computer is configured with the MicroNIR Pro software system, tailored specifically for managing and analyzing spectral data. This software system serves as a repository for storing the acquired spectral data of seed cotton. It plays a pivotal role in safeguarding both raw and processed spectral data. When gathering spectral data from seed cotton, every sample was uniformly distributed across the base of the container. Spectral measurements were taken at five distinct points on the seed cotton (depicted as red circular zones in **Figure 1**) utilizing the JDSU near-infrared spectrometer. This approach enabled the acquisition of five individual sets of spectral data for each sample, subsequently allowing the computation of the mean value derived from these five datasets. The resulting average value was then utilized as the representative spectral data for each individual sample.

### 2.3 Dataset preparation

The appropriateness of sample set division significantly influences the subsequent reliability of constructed machine learning or deep learning models. An imbalanced distribution between training and test sets can significantly impact the reliability of modeling outcomes. If the training set comprises an excessive proportion of samples while the test set remains disproportionately small, the representation of test set samples becomes inadequate, thereby diminishing the credibility of modeling results obtained from the test set. Conversely, an inadequate representation of training set samples can lead to an underfitting of the model. Therefore, achieving a balanced allocation of samples between the training and test sets is pivotal in ensuring dependable and accurate modeling outcomes. The SPXY (Sample Set Partitioning Based on Joint X-Y Distance) algorithm operates by segregating samples into these sets through a rigorous assessment of their Euclidean distances within both the x and y dimensions (Chen et al., 2021). Hence, within the scope of this study, a total of 230 seed cotton spectral data points were meticulously partitioned into distinct training and test sets through the application of the SPXY algorithm. Precisely, the training set encompasses 180 seed cotton samples, while the test set consists of 50 seed cotton samples.

### 2.4 Pretreatment of spectral data

During the experimental process, the considerable interstices among the seed cotton inherently contribute to light scattering. Additionally, variations in temperature and the presence of light scattering, both inherent to the experimental conditions, contribute to the introduction of noise into the seed cotton spectral data. The existence of such noise inherently undermines the development of subsequent spectral analysis models (Mishra and Lohumi, 2021). To mitigate the impact of this noise, this study employed the utilization of Normalization, Savitzky-Golay convolutional smoothing (SG), and Standard Normal Variate Transformation (SNV) techniques. These methods were employed to effectively counteract the introduced noise and thereby enhance the reliability of the subsequent analysis. The objective of normalization is to achieve an isometric scaling of eigenvalues (Wang et al., 2020). This is accomplished by subtracting the minimum value of the sample data from each data point and subsequently dividing it by the range between the maximum and minimum values of the sample. This process effectively maps all data points into the interval (0, 1). The normalization formula is denoted as Equation 1 and is expressed as follows.


(1) $$x_{1}=\frac{x-\min(x)}{\max(x)-\min(x)}$$

The fundamental principle underlying the SG algorithm entails the initial establishment of a window size capable of accommodating a specific quantity of data points. This window size facilitates the determination of the central data point within the window. Subsequently, all data points encompassed by the window are subjected to polynomial fitting, a process adept at retaining pertinent spectral information while concurrently effecting smoothing. This methodology effectively balances the preservation of essential spectral details with the achievement of a smoothing effect (Pokhrel et al., 2023). The SNV algorithm serves the purpose of normalizing an individual spectrum in order to mitigate the influence of scattering arising from particles situated on the surface of the seed cotton (Qi et al., 2022). The SNV algorithm is executed using the following formula, referred to as Equation 2.


(2) $$y=\frac{x_{i}-\bar{x}}{\sqrt{\frac{\sum_{i=1}^{n}(x_{i}-\bar{x}{)}^{2}}{n-1}}}$$

This formula y represents the outcome of a specific spectrum subsequent to undergoing SNV processing. $x_{i}$ signifies the value of the ith data point within this spectrum. $\bar{x}$ represents the mean value derived from all the data points within the given spectrum, n represents the total count of wavelength points.

### 2.5 Data processing methods based on machine learning

#### 2.5.1 Feature selection for spectral data

The acquired seed cotton near-infrared spectral data in this study encompass a dataset spanning 125 dimensions. This voluminous dataset, in conjunction with a limited sample size of only 230, presents a susceptibility to overfitting when employing conventional machine learning algorithms that do not integrate dimensionality reduction techniques (Xiao et al., 2022). To circumvent this challenge, the study employed two distinct methodologies, namely the successive projections algorithm (SPA) and the Random Frog (RF) algorithm. These techniques were employed to identify feature wavelengths within the seed cotton spectral data. This selection process aimed to identify a subset of wavelength points that could effectively and accurately predict the impurity content of the seed cotton.

The SPA is a forward variable selection algorithm that initiates with a specific wavelength variable. In each iteration, it computes the projection of this variable onto the remaining wavelengths. This cyclic process facilitates the reduction of covariance between variables, resulting in the elimination of redundant information while retaining valuable insights. The outcome is an enhancement in both the predictive accuracy and efficiency of the model (Ning et al., 2022). Following the application of the SPA algorithm, irrelevant information is effectively pruned, thereby enabling the algorithm to identify the feature wavelengths. Within the context of the RF algorithm, the iterative computational procedure unfolds through three primary stages for a spectral variable *X*. Here, the *n* rows correspond to the sample count, the *p* columns denote the variables, and the corresponding target matrix *Y* is composed of *n×1* variables (Zhang et al., 2023). The sequence of steps is as follows:

Random subset generation: An initial subset *V_0_* comprising *Q* variables is generated randomly.

Candidate subset proposition: A subset V* of candidate variables, encompassing Q* variables, is proposed based on *V_0_*. V* is accepted as *V1* with a certain probability. Subsequently, *V_0_* is replaced by *V_1_* and the process iterates.

Variable importance evaluation: The algorithm calculates the probability value for each variable selection, thereby serving as an indicator of the assessment of variable importance.

#### 2.5.2 Modeling methods

This study employed partial least squares regression (PLSR), support vector regression (SVR), and least squares support vector machine (LSSVM) methodologies to construct regression prediction models aimed at forecasting the impurity content within seed cotton.

**Figure 2:**
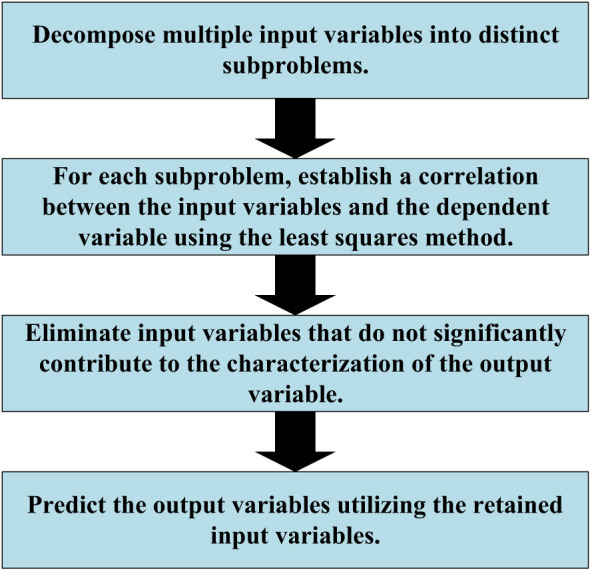
PLSR.

PLSR is a methodology employed to elucidate the linear interplay between input variables and output variables. Its fundamental process encompasses the subsequent steps (Shetty and Gislum, 2011), as illustrated in **Figure 2**:

1. Decomposition of relationships: The intricate connection between multiple input variables and a singular output variable is divided into numerous subproblems. Each subproblem corresponds to the relationship between an individual input variable and the output variable.
2. Characterization via Least Squares: For each subproblem, the association between input variables and the dependent variable is established through the least squares method, enabling a refined understanding of the interaction.
3. Variable significance evaluation: The contribution of each input variable to the output variable is assessed, guiding the decision to retain or eliminate variables based on their statistical significance. Non-significant input variables are progressively removed through an iterative process, resulting in a streamlined model.
4. Prediction and model evaluation: Utilizing the simplified model, predictions for output variable values are generated. The model’s performance is subsequently assessed through the evaluation of prediction errors.

The fundamental tenet of SVR revolves around minimizing the discrepancy between predicted and actual outcomes through the identification of an optimal hyperplane, facilitating the mapping of input data to output data. This process encompasses the subsequent stages:

a. The input data is projected into a high-dimensional feature space.
b. A hyperplane is constructed within this feature space, serving as the foundation for making predictions and facilitating the regression task.
c. The process involves the identification of support vectors—data points lying closest to the hyperplane within the feature space. These vectors are pivotal in determining the position of the hyperplane.
d. The optimization of hyperplane parameters is achieved by minimizing a designated objective function. The minimization objective function is as Equation 3.

(3) $$\min\frac{1}{2}{∥w∥}^{2}+C\sum_{i=1}^{n}\max(0,y_{i}-w^{T}\Phi_{i}-b)$$

This formula w signifies the normal vector of the hyperplane, while b represents the intercept. $\Phi_{i}$ corresponds to the “ith” sample point within the input space, and $y_{i}$ denotes the output value range associated with that particular sample point. The notation encapsulates the range of possible values for the output value at the said sample point. The parameter C assumes the role of a regularization parameter, responsible for governing the extent of variation exhibited by the slack variable $x_{i}$ .

The core principle of LSSVM shares similarities with SVR, involving the projection of input data into a higher-dimensional space and the determination of an optimal hyperplane to facilitate output data prediction. However, a notable distinction lies in their respective error fitting mechanisms. In LSSVM, error fitting is executed through the least squares method, while SVR achieves error fitting by leveraging the support vectors. This difference underscores the unique approach that LSSVM employs in aligning the model with the training data, contributing to its effectiveness in predicting output values within the context of seed cotton impurity content analysis (Luo et al., 2023).

### 2.6 Cotton-Net

**Figure 3:**
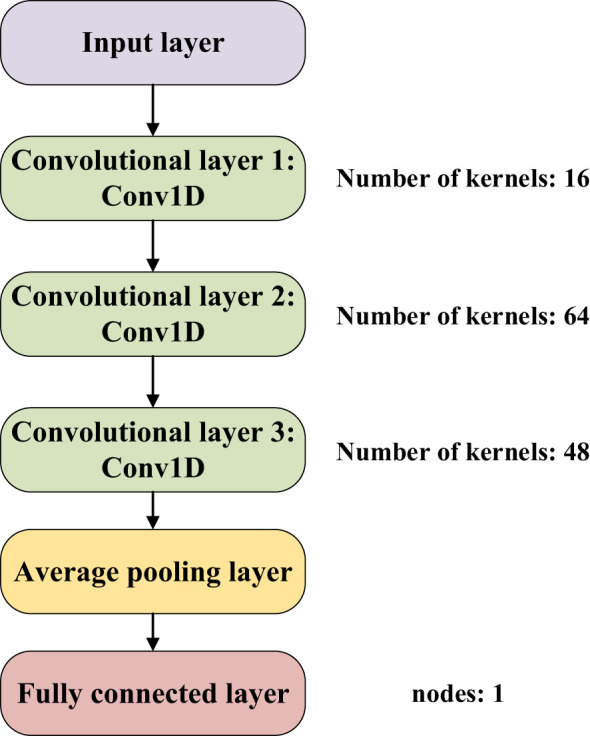
Cotton-Net.

Convolutional neural networks possess robust feature extraction capabilities and have witnessed successful applications within spectroscopy in recent years (Yuan et al., 2022). A case in point is the work by Yang et al. (2021), where a convolutional neural network in conjunction with near-infrared spectroscopy was effectively employed to discern the source of tea. This study involved a substantial dataset of 480 samples. Similarly, Li et al. (2023) harnessed the power of CNNs coupled with spectroscopy to identify the provenance of duck eggs, utilizing a dataset encompassing 261 samples. Furthermore, Hu et al. (2023) applied a one-dimensional convolutional neural network in conjunction with spectroscopy to detect pesticide residues on the Hami melon surface. This endeavor incorporated a dataset containing 200 samples. Incorporating 230 seed cotton samples into a dataset, the application of convolutional neural networks arises as a viable method for the automated extraction of seed cotton-specific spectral features. The unique attributes of the seed cotton spectral data, encompassing a dimensionality of 1×125, form the foundation of this study. Through iterative refinements, the construction of a convolutional neural network architecture referred to as “Cotton-Net”, was achieved as depicted in **Figure 3**.

As illustrated in **Figure 3**, the architecture of Cotton-Net comprises a total of 6 layers, incorporating 3 one-dimensional convolutional layers, 1 average pooling layer, and 1 fully connected layer. The initial convolutional layer is equipped with 16 convolution kernels, the second layer integrates 64 kernels, and the third employs 48 kernels. This design is strategically aimed at extracting the salient features inherent to seed cotton spectral data. The introduction of an average pooling layer serves the purpose of streamlining network computations while also mitigating the risk of model overfitting. Lastly, the fully connected layer contributes to the ultimate prediction of seed cotton impurity content, with a single node dedicated to this prediction outcome. This comprehensive architectural arrangement within Cotton-Net facilitates the intricate analysis of seed cotton spectral data, resulting in precise predictions.

The loss function serves as a metric for quantifying the disparity between the model’s predictions and actual values, thereby driving the continuous refinement of the network’s parameters. The selection of an appropriate loss function holds paramount importance, as it profoundly influences both model convergence and performance outcomes. Within the context of this investigation, the chosen loss function for Cotton-Net is the mean squared logarithmic error. The formula for the realization of the mean squared logarithmic error is denoted as Equation 4.


(4) $$MSLE=\log(\frac{\sum_{i=1}^{n}{\left(\hat{y}-y\right)}^{2}}{n})$$

This formula, n represents the total count of samples under consideration. y corresponds to the actual impurity content value of the seed cotton sample, while $\hat{y}$ symbolizes the network’s predicted impurity content value for the same seed cotton sample.

### 2.7 Performance evaluation of models

Frequently employed metrics for assessing the efficacy of regression models encompass the correlation coefficient and root mean square error. Typically, higher values approaching 1 for the correlation coefficient, alongside a smaller root mean square error nearing 0, indicate enhanced predictive prowess and model stability (Fan et al., 2020). These metrics collectively provide insights into the model’s capacity to accurately capture relationships and deliver reliable predictions within the regression context. Their formulas are as follows: Equations 5 and 6.


(5) $$R=\frac{\sqrt{\sum_{i=1}^{n}{\left({\hat{y}}_{i}-y_{i}\right)}^{2}}}{\sqrt{\sum_{i=1}^{n}{\left({\hat{y}}_{i}-y_{mean}\right)}^{2}}}$$

(6) $$RMSE=\sqrt{\frac{1}{n}\sum_{i=1}^{n}{\left({\hat{y}}_{i}-y_{i}\right)}^{2}}$$

This formula, n denotes the count of samples within the dataset. ${\hat{y}}_{i}$ signifies the predicted value for the “ith” sample, $y_{i}$ represents the actual value of the same “ith” sample. Additionally, $y_{mean}$ stands for the mean value computed from the actual values across all samples encompassed by the dataset.

## 3 Results and discussion

### 3.1 Model result analysis based on machine learning

#### 3.1.1 Sensitive band analysis of seed cotton

**Figure 4:**
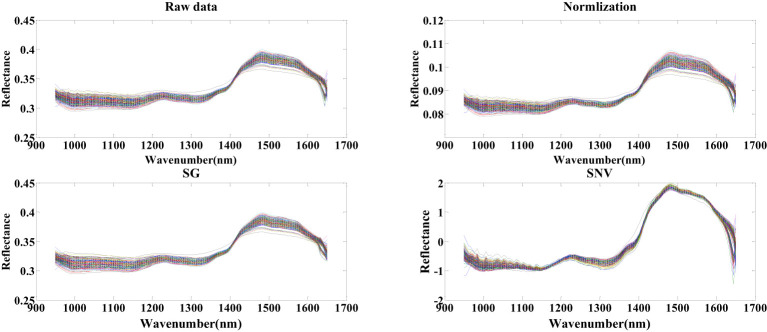
Spectral data of seed cotton before and after the pretreatment.

Prior to the selection of seed cotton’s spectral feature wavelength points, pretreatment procedures involving SG, SNV, and normalization were conducted on the spectral data. As illustrated in **Figure 4**, a comparison is presented between the raw seed cotton spectral data and the data that underwent pretreatment using the three distinct methods. Notably, the distributions of seed cotton spectral data remain consistent with the raw dataset after both normalization and SG pretreatment. However, a slight alteration in data distribution is observed following SNV pretreatment. In contrast to the raw data, the reflectance of seed cotton spectral data after normalization pretreatment exhibited normalization within the range of 0.05 to 0.11. The SG pretreatment resulted in a comparatively smoother spectral profile, and the SNV pretreatment led to a normalization of spectral data within the range of -2 to 2.

**Figure 5:**
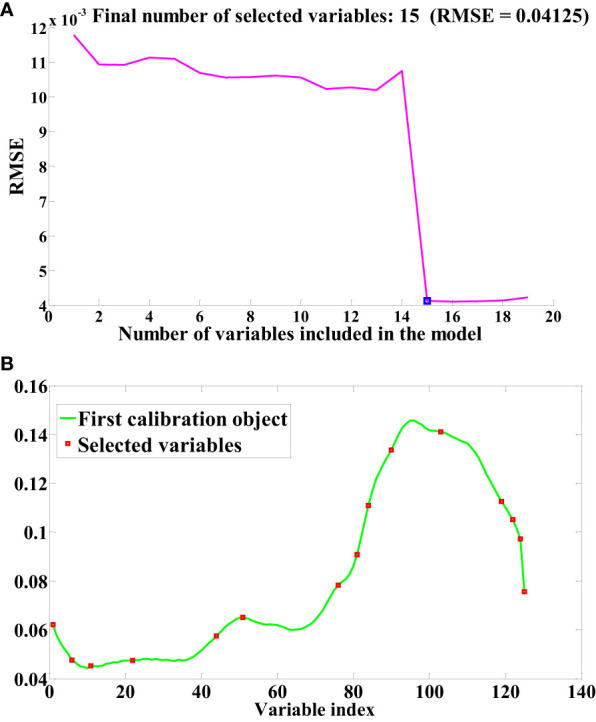
Sensitive bands selection based on SPA. **(A)** RMSE **(B)** Index of selected variables.

The SPA and RF algorithms were employed to select feature wavelengths from the pretreated seed cotton spectral data. As depicted in **Figure 5**, the outcomes of the feature wavelength selection performed by the SPA algorithm are presented (In this study, Normalization pretreatment is taken as an example to elucidate the process of feature wavelength selection using SPA). The fundamental principle governing the SPA algorithm for feature wavelength selection in seed cotton involves minimizing the root mean square error to identify the pivotal point with the smallest such error. This process effectively retains pertinent information and discards redundancy leading up to the pivotal point. As evident in **Figure 5A**, the SPA algorithm has discerningly chosen 15 feature wavelength points capable of accurately reflecting the impurity content within the seed cotton samples. **Figure 5B** illustrates the distribution of feature wavelength points for seed cotton, as selected through the SPA algorithm. The selection of feature wavelength points following SG and SNV pretreatment mirrored that of Normalization. Following SG pretreatment, the final number of selected feature wavelength points is 17, while after SNV preprocessing, it is 34.

**Figure 6:**
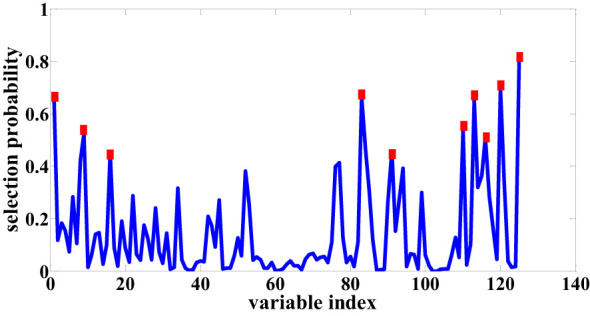
Sensitive bands selection based on RF.

The RF algorithm operates iteratively, systematically reducing variables and assigning a probability score to each variable’s potential for selection, ultimately achieving the task of feature wavelength selection for seed cotton (Once more, the seed cotton data subjected to Normalization pretreatment is employed to illustrate the procedure of feature wavelength selection using the RF algorithm.). In this research, through meticulous experimentation, parameters were optimized: iterations were set at 1000, the initial data subset comprised 5 variables, and the chosen data preprocessing approach was “center”. These parameters collectively contributed to the effective execution of the RF algorithm in identifying key spectral characteristics pertinent to seed cotton analysis. The outcomes of the selected feature wavelength points for seed cotton are visually presented in **Figure 6**. Similarly, the reuse RF algorithm selects 8 feature wavelength points after SG preprocessing and 9 after SNV pretreatment.

#### 3.1.2 Regression prediction based on PLSR, SVR, LSSVM

Upon completing the selection of feature wavelengths within the seed cotton spectral data, separate models for predicting seed cotton impurity content were constructed using PLSR, SVR, and LSSVM methodologies. In this research, the PLSR model employed 4 principal components, while the SVR and LSSVM models adopted the radial basis function. The modeling outcomes of PLSR, SVR, and LSSVM are illustrated in **Tables 1**–**3**, respectively. For the PLSR model, the optimal performance was attained by employing Normalization pretreatment followed by feature wavelength point selection through SPA. This configuration yielded a test set correlation coefficient of 0.8001 and an RMSE of 0.0738. Similarly, in the case of the SVR model, the most favorable results were achieved by applying Normalization preprocessing in conjunction with SPA for feature wavelength point selection. This configuration yielded the best performance metrics, showcasing a test set correlation coefficient of 0.7512 and an RMSE of 0.0766. Regarding the LSSVM model, the most superior performance was achieved by employing Normalization preprocessing followed by the RF algorithm for feature wavelength point selection. This specific configuration showcased the optimal performance metrics, including a test set correlation coefficient of 0.8662 and an RMSE of 0.0622.

**Table 1:** The results of PLSR.

Pre-Processing	VN	R_C_	RMSE_C_	R_P_	RMSE_P_
SG + SPA	17	0.9172	0.0932	0.7191	0.0885
SG + RF	8	0.8845	0.1091	0.7023	0.0944
Normalization + RF	10	0.8931	0.1054	0.7698	0.0787
Normalization + SPA	15	0.9154	0.0946	0.8001	0.0738
SNV + RF	9	0.5266	0.1985	0.6584	0.0955
SNV + SPA	34	0.7733	0.1483	0.5941	0.0983

R_C_, Correlation coefficient of the training set; RMSE_C_, RMSE of the training set; R_P_, Correlation coefficient of the test set; RMSE_P_, RMSE of the test set; VN, number of variables.

**Table 2:** The results of SVR.

Pre-Processing	VN	R_C_	RMSE_C_	R_P_	RMSE_P_
SG + SPA	17	0.8216	0.1674	0.6877	0.0932
SG + RF	8	0.8649	0.1243	0.7023	0.0853
Normalization + RF	10	0.8735	0.1215	0.7286	0.0833
Normalization + SPA	15	0.8864	0.1156	0.7512	0.0766
SNV + RF	9	0.7598	0.1594	0.6866	0.0869
SNV + SPA	34	0.9384	0.0853	0.7220	0.0846

R_C_, Correlation coefficient of the training set; RMSE_C_, RMSE of the training set; R_P_, Correlation coefficient of the test set; RMSE_P_, RMSE of the test set; VN, number of variables.

**Table 3:** The results of LSSVM.

Pre-Processing	VN	R_C_	RMSE_C_	R_P_	RMSE_P_
SG + SPA	17	0.9548	0.0693	0.8577	0.0684
SG + RF	8	0.9690	0.0586	0.7972	0.0791
Normalization + RF	10	0.9571	0.0685	0.8662	0.0622
Normalization + SPA	15	0.9667	0.0638	0.8543	0.0696
SNV + RF	9	0.8575	0.1304	0.7094	0.0861
SNV + SPA	34	0.9472	0.0712	0.8578	0.0683

R_C_, Correlation coefficient of the training set; RMSE_C_, RMSE of the training set; R_P_, Correlation coefficient of the test set; RMSE_P_, RMSE of the test set; VN, number of variables.

### 3.2 Model result analysis based on Cotton-Net

#### 3.2.1 Experimental configuration

The hardware and software framework employed for Cotton-Net training comprised an Intel i9-12900K CPU, NVIDIA GeForce RTX 3090Ti GPU, Pytorch 1.12 as the deep learning framework, and CUDA 11.7 as the computing platform. The procedure of training Cotton-Net involves refining the model’s prediction of seed cotton impurity content to progressively align with the actual values. In this research, the network’s parameters were optimized through the utilization of the Adam optimizer, initialized with a learning rate of 0.001. The training process was executed for a maximum of 200 epochs, with a batchsize set at 8.

#### 3.2.2 Ablation experiments based on different activation functions

**Figure 7:**
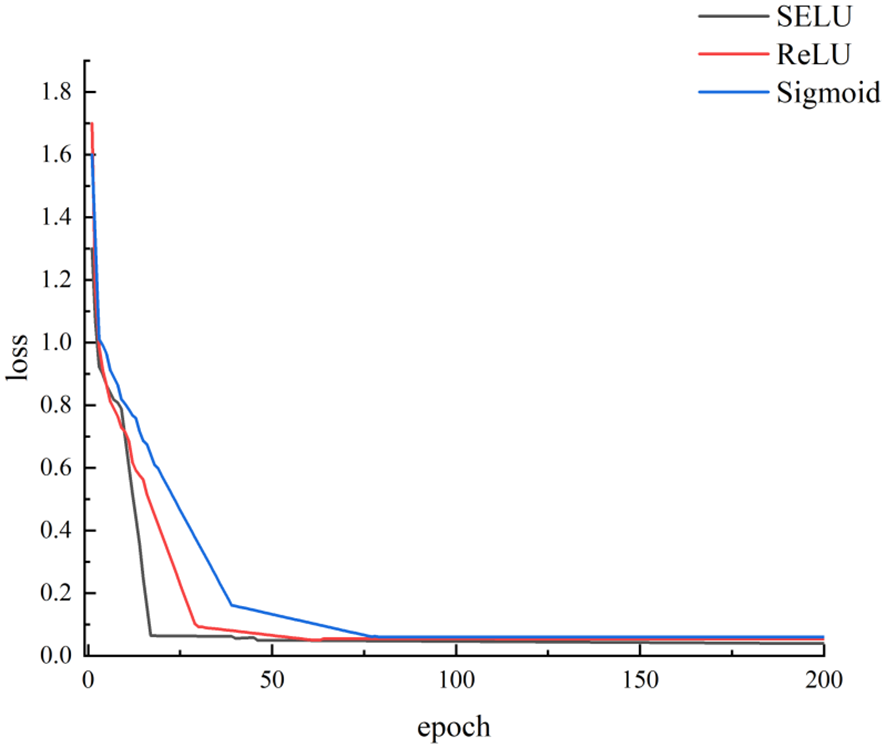
Loss curves with different activation functions.

The selection of an appropriate activation function within convolutional neural networks significantly impacts network performance. Opting for a well-suited activation function not only facilitates swift convergence of the network but also enhances model prediction performance. In this research, we explored the implementation of Sigmoid, ReLU, and SELU as potential activation functions for Cotton-Net. The input for Cotton-Net comprised seed cotton full-spectrum data subjected to SG, Normalization, and SNV pretreatment. **Figure 7** illustrates the progression of loss curves during the training of Cotton-Net with three distinct activation functions (utilizing Normalization as the pretreatment method). Notably, it becomes apparent that the network employing the SELU activation function demonstrated the swiftest convergence rate, while the network adopting the Sigmoid activation function exhibited a more gradual convergence. Ultimately, all three networks attained and maintained low loss values, underscoring the model’s efficacy in predicting seed cotton impurity content.

Ablation experiments were conducted employing three distinct activation functions on seed cotton spectral data following different preprocessing methodologies. The results of these experiments are detailed in **Table 4**. It is evident that when utilizing SELU as the activation function in Cotton-Net, higher correlation coefficients between predicted and actual impurity content values for seed cotton samples in the test set were achieved compared to employing ReLU and Sigmoid as the activation functions. Notably, among these experiments, the Cotton-Net with Normalization pretreatment exhibited the most exceptional predictive performance. It achieved a noteworthy correlation coefficient of 0.9063 and an RMSE of 0.0546 for predicting test set samples. Furthermore, the correlation coefficients for the Cotton-Net’s predictions of the test set samples consistently exceeded 0.8. This observation suggests that the overall predictive performance of Cotton-Net, in terms of seed cotton impurity content, surpasses that of PLSR. Thus, the predictive capabilities of Cotton-Net prove to be superior compared to those of PLSR, SVR, and LSSVM.

**Table 4:** Ablation experiment results.

Model	R_C_	RMSE_C_	R_P_	RMSE_P_
SG + SELU	0.9216	0.0928	0.8236	0.0724
SG + ReLU	0.9158	0.0944	0.8017	0.0735
SG + Sigmoid	0.9237	0.0913	0.8124	0.0731
Normalization + SELU	0.9683	0.0592	0.9063	0.0546
Normalization + ReLU	0.9582	0.0657	0.8561	0.0679
Normalization + Sigmoid	0.9534	0.0699	0.8487	0.0713
SNV + SELU	0.9579	0.0658	0.8642	0.0668
SNV + ReLU	0.9325	0.0874	0.8157	0.0722
SNV + Sigmoid	0.9514	0.0701	0.8437	0.0716

### 3.3 Comparison of optimal models

**Figure 8:**
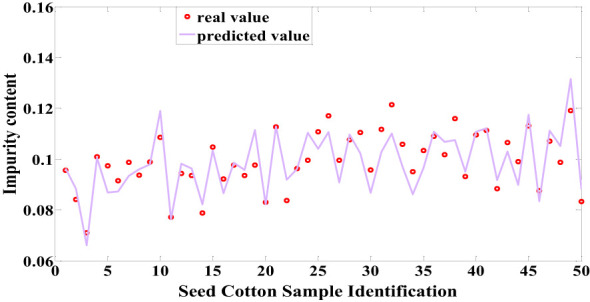
Predicted values of LSSVM.

**Figure 9:**
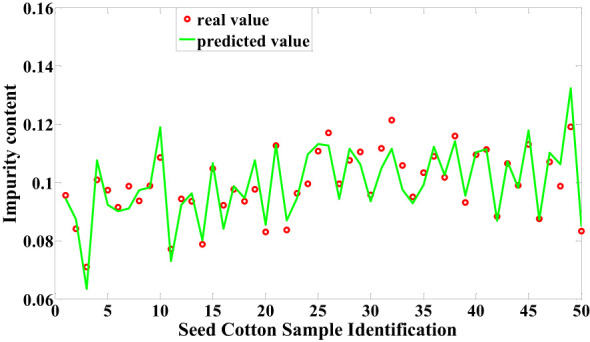
Predicted values of Cotton-Net.

In this study, both machine learning techniques and Cotton-Net were employed for predicting the impurity content in machine-picked seed cotton. Among the machine learning models, the LSSVM model constructed following Normalization + RF processing demonstrated the highest performance in predicting the impurity content of seed cotton samples within the test set. This model achieved an impressive correlation coefficient of 0.8662 and an RMSE of 0.0622, as depicted in **Figure 8**. On the other hand, the most optimal performance achieved by Cotton-Net for predicting the impurity content of seed cotton samples in the test set was attained by the model constructed after Normalization preprocessing, with SELU used as the activation function. Notably, this model showcased a remarkable correlation coefficient of 0.9063 and an RMSE of 0.0546, as presented in **Figure 9**. The comparison reveals that Cotton-Net surpasses machine learning in predicting the impurity content of seed cotton.

## 4 Discussion

To expedite the impurity content detection in machine-picked seed cotton, this study established a seed cotton spectral data acquisition system using near-infrared spectroscopy. Leveraging the characteristics of seed cotton spectral data, a one-dimensional convolutional neural network, known as Cotton-Net, was constructed for impurity content prediction in machine-picked seed cotton. Various researchers have undertaken investigations into cotton impurity detection. For instance, Xu et al. (2023) employed machine vision to classify impurities in lint cotton, achieving an accuracy of 98%. In contrast, the task of seed cotton impurity detection undertaken in this study posed notably greater challenges than lint cotton. Nevertheless, successful prediction of seed cotton impurity content was attained. Similarly, Chang et al. (2021) harnessed hyperspectral technology to classify impurities in seed cotton, yielding a classification accuracy of 83.40%, yet the prediction of impurity content was not realized. Zhang et al. (2021; Zhang et al., 2022) utilized machine vision to predict impurity content in seed cotton, albeit achieving a low prediction accuracy of 0.8. In comparison, the present study accomplished a higher level of impurity detection. Hence, the approach employed in this study proves to be more suitable for seed cotton impurity detection in contrast to the aforementioned investigations.

## 5 Conclusions

The following conclusions can be inferred:

1. The experimental outcomes revealed that utilizing SELU as the activation function enhances both the convergence speed and the model performance of Cotton-Net. Among these experiments, the Cotton-Net model after Normalization preprocessing exhibited the most favorable predictive performance, showcasing a correlation coefficient of 0.9063 and an RMSE of 0.0546.
2. Adopting machine learning algorithms, the LSSVM model built after Normalization pretreatment and employing the RF algorithm for feature wavelength selection demonstrated the optimal performance, with a correlation coefficient of 0.8662 and an RMSE of 0.0622. Comparatively, the performance of Cotton-Net exhibited significant improvement.

## Data availability statement

The original contributions presented in the study are included in the article/supplementary material. Further inquiries can be directed to the corresponding author.

## Author contributions

QL: Data curation, Formal Analysis, Investigation, Methodology, Validation, Visualization, Writing – original draft. WZ: Conceptualization, Funding acquisition, Project administration, Resources, Writing – review & editing. XZ: Investigation, Methodology, Writing – review & editing. HL: Software, Writing – review & editing. ML: Writing – review & editing. HJL: Writing – review & editing.
